# Regenerative Approaches in Vulvar Lichen Sclerosus: A Systematic Review

**DOI:** 10.3390/ijms26188808

**Published:** 2025-09-10

**Authors:** Katarzyna Beutler, Alina Jankowska-Konsur, Danuta Nowicka

**Affiliations:** 1 University Centre of General Dermatology and Oncodermatology, Wroclaw Medical University, 50-368 Wroclaw, Poland; 2 Division of Aesthetic Dermatology and Regenerative Medicine of the Skin, Wroclaw Medical University, 50-368 Wroclaw, Poland

**Keywords:** vulvar lichen sclerosus, mesotherapy, alternative treatment, emerging therapy

## Abstract

Vulvar lichen sclerosus (VLS) involves chronic inflammation, immune dysregulation, and abnormal extracellular matrix remodeling, involving extracellular matrix protein 1 (ECM1) and non-coding RNAs, particularly miR-155. Platelet-rich plasma (PRP) and adipose-derived mesenchymal stem cells (ADSCs) offer regenerative potential through the release of growth factors and cytokines that promote angiogenesis, fibroblast proliferation, collagen synthesis, and tissue repair, which could potentially compensate for the disordered matrix in VLS. This systematic review aimed to evaluate the current evidence on the efficacy and safety of PRP, ADSCs, and active substances administered through mesotherapy to adult women with VLS. A search of the PubMed, Scopus, and Web of Science databases identified 251 records, of which 13 studies met the inclusion criteria (RCTs and cohort studies involving women aged ≥ 18 years who were treated with PRP, ADSCs, or mesotherapy). The reviewed studies suggest that these therapies may improve clinical symptoms, quality of life, sexual function, and tissue quality. However, their application may be constrained by procedural invasiveness and potential immunologic risks. Moreover, the current evidence base is limited by small sample sizes, a lack of control groups, and short follow-up periods. Larger, well-designed randomized controlled trials with long-term follow-up are needed to confirm their therapeutic value and establish clear clinical guidelines.

## 1. Introduction

Vulvar lichen sclerosus (VLS) is a chronic inflammatory skin condition that primarily affects the genital area and may lead to sexual dysfunction [1]. Its exact cause remains unknown, and both its etiology and pathogenesis are not yet fully understood. However, there is evidence of genetic and familial predisposition [2]. Contributing factors may include repeated trauma, hormonal influences, and certain medications. VLS is considered an immune-mediated disease driven by T helper type 1 (Th1) cells and dependent on microRNA-155 (miR-155). Although autoantibodies targeting extracellular matrix protein 1 (ECM1) and BP180 have been identified in some cases, their precise role in the disease process remains uncertain. Additionally, VLS is associated with the abnormal expression of genes involved in tissue remodeling and elevated oxidative stress, both of which may contribute to scarring and an increased risk of malignancy [3,4,5].

VLS most commonly affects women in their 40s and 50s, but it can occur at any age [6,7]. VLS shows a bimodal distribution, with peaks in prepubertal girls and postmenopausal women [8]. Men can be affected as well, though significantly less often than women. VLS is newly diagnosed in approximately 0.1–0.3% of patients in general hospital settings and in about 1.7% of patients referred to general gynecology clinics. However, the exact prevalence and incidence are not known. The scale of this illness is likely to be underestimated because of a reluctance to discuss genital symptoms with their physicians and because some women with VLS are either asymptomatic or experience only mild symptoms [7].

In 2024, experts identified important clinical features of VLS using an international electronic Delphi consensus. The group reached a consensus on five critical diagnostic features for VLS in adults, along with an additional 12 features considered potentially important [7,9]. The five key diagnostic features identified by both experts and patients were white discoloration of the skin, itching, anatomical changes, burying of the clitoral area, and noticeable improvement with topical steroid treatment. The 12 diagnostic features of VLS that were considered important but not critical were identified in descending order of perceived importance. These included crinkly skin, bruising or bleeding under the skin, fissuring at the posterior vaginal entrance, and absence of vaginal involvement. Other features noted were erosions, a loss of skin elasticity, irritation, and skin thickening. Pain or soreness related to sexual activity, perianal fissures, and pain or soreness unrelated to sexual activity were also recognized among these features [7,10].

VLS is often overlooked or incorrectly diagnosed, leading to delays in appropriate treatment and the development of complications [7]. If left untreated, VLS can lead to substantial and irreversible scarring and distortion of the vulvar anatomy. Moreover, it carries a 2–6% lifetime risk of developing malignant squamous neoplasia of the vulva. This increased risk of progression to vulvar squamous cell carcinoma represents a serious potential complication [6]. Additionally, distressing symptoms experienced by affected patients reduce health-related quality of life [11].

A timely diagnosis enables the initiation of effective treatment, which can prevent scarring, lower the risk of malignancy, and help preserve the good quality of life of affected patients [7]. High-potency topical corticosteroids continue to be the gold standard of treatment and first-line treatment [12]. Follow-up appointments are typically scheduled every three to six months during the first two years and at least once a year thereafter to monitor treatment effectiveness and promote adherence to the management plan. Long-term monitoring is recommended due to the chances of recurrence, persistence of symptoms, and development of adverse events of treatment [2,8]. In response to the insufficiencies of standard therapy, alternative treatments that can alter the immune system and encourage tissue regeneration are gaining popularity [13,14]. Therapies using platelet-rich plasma (PRP), adipose-derived mesenchymal stem cells (ADSCs), and various active substances administered by mesotherapy techniques are attracting particular attention [15,16,17,18]. These methods, although still at the stage of research or experimental applications, show potential in the context of recovery of vulvar tissue integrity and elasticity and alleviating disease symptoms [8]. Injections of PRP and ADSCs trigger a cascade of cellular and molecular processes that underlie their therapeutic effects on VLS. While the exact pathogenesis of VLS is not fully understood, key mechanisms include immune system dysregulation, chronic inflammation, and impaired angiogenesis [19]. These are accompanied by increased fibroblast activity and abnormal collagen production, which contribute to the progressive formation of thickened, sclerotic skin. The biological actions of PRP and ADSCs target these processes, supporting their potential in treating VLS-related lesions [4]. Oyama et al. [20] found that patients with lichen sclerosus had IgG autoantibodies in their plasma and exhibited a specific humoral immune response to ECM1, a protein found in the skin. ECM1 plays a role in epidermal differentiation by binding to dermal matrix components such as perlecan and contributes to the stimulation of vascular endothelial cells within the tumor stroma. In addition, Han et al. [21] discovered that ECM1 plays a role in angiogenesis by selectively stimulating the proliferation of vascular endothelial cells. Similarly, PRP delivers a range of growth factors that promote fibroblast proliferation, collagen synthesis, and tissue remodeling [22]. ADSCs have similar effects, as they secrete a variety of paracrine factors, including growth factors and cytokines, that support angiogenesis, stimulate fibroblast proliferation, enhance collagen synthesis, and promote tissue remodeling. Anti-inflammatory cytokines such as interleukin-10 and TGF-β also help reduce excessive inflammation and facilitate extracellular matrix deposition [23]. Although neither PRP nor ADSCs directly target ECM1, they may support the regeneration of the extracellular matrix that is disrupted due to ECM1 dysfunction. By enhancing fibroblast activity and promoting collagen remodeling, these therapies could help restore structural integrity and potentially compensate for the impaired matrix seen in VLS.

**Figure 1:**
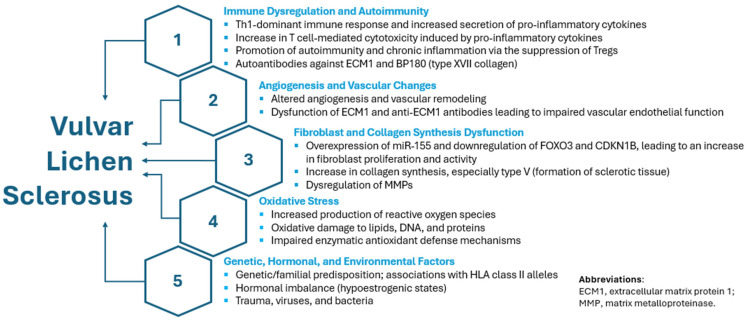
Pathophysiological mechanisms contributing to the development of VLS.

Furthermore, miR-155 is a pro-inflammatory microRNA that plays a key role in regulating immune responses, including T-cell differentiation and the production of inflammatory cytokines [24]. Its overexpression leads to the downregulation of FOXO3 and CDKN1B, which promotes fibroblast proliferation. Consequently, miR-155 may contribute to the development of VLS by driving both autoimmunity and excessive fibroblast activity [4]. ADSCs can secrete exosomes containing various non-coding RNAs, including microRNAs that influence immune responses [23]. These exosomes, which also carry proteins and lipids, have been shown to regulate inflammation; however, the balance between their pro- and anti-inflammatory effects remains unclear [25]. Notably, miR-155 expression is upregulated in response to multiple inflammatory stimuli, including TNF-α, IL-1β, interferons, pathogen-associated and damage-associated molecular patterns, and alarmins such as IL-1α [24]. The main pathophysiological mechanisms of VLS are depicted in Figure 1.

This review aimed to evaluate the efficacy and potential benefits of modern and innovative treatments for VLS, with a particular focus on biologic and regenerative therapies such as PRP, ADSC transplants, and active substance mesotherapy. It attempted to present the current state of knowledge regarding these approaches, assess their effectiveness, and explore their roles in complementing or potentially replacing conventional treatment options.

## 2. Materials and Methods

The search was conducted on 16th March 2025 according to the Preferred Reporting Items for Systematic Reviews and Meta-Analyses (PRISMA) statement guidelines [26] using the databases PubMed, Scopus, and Web of Science. The search formulas included the following:

For PubMed, (lichen sclerosus OR vulvar lichen sclerosus) AND (PRP OR platelet-rich plasma OR mesotherapy OR stem cells)—51 documents;

For Scopus, TITLE-ABS-KEY (“lichen sclerosus” OR “vulvar lichen sclerosus”) AND (PRP OR “platelet-rich plasma” OR mesotherapy OR “stem cells”)—116 documents;

For Web of Science, TS = (lichen sclerosus OR vulvar lichen sclerosus) AND (PRP OR platelet-rich plasma OR mesotherapy OR stem cells)—84 documents.

Records were screened by the title, abstract, and full text by one investigator. Two independent researchers were involved in both the literature search and the data extraction processes, with disagreements resolved by the third senior researcher. Studies included in this review matched all the predefined criteria according to PI(E)COS (“Population”, “Intervention”/”Exposure”, “Comparison”, “Outcomes”, and “Study design”) [27], as shown in Table 1. A detailed search flowchart is presented in Section 3.

## 3. Results

**Figure 2:**
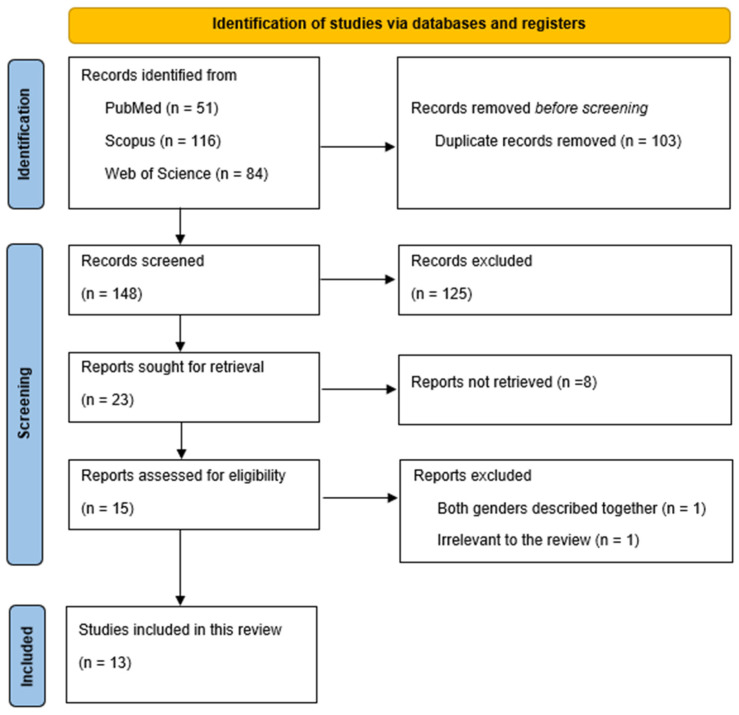
PRISMA flowchart of the selected studies.

In the search, we identified 251 records (51 in PubMed; 116 in Scopus; and 84 in Web of Science). Out of these, 103 duplicate records were removed. A total of 148 titles and abstracts were screened, and out of these, 125 were excluded. Twenty-three articles were sought for retrieval and 22 were assessed for eligibility. Thirteen articles met the inclusion criteria and were summarized in this review. The selection process is presented in the PRISMA flowchart (Figure 2).

The studies included in this review were conducted between 2015 and 2024. Studies were conducted in the USA (2), Italy (6), Spain (2), Australia (1), Greece (1), and the United Kingdom (1). These studies included a total of 360 women diagnosed with VLS. In total, one out of the thirteen studies was reported as a randomized controlled trial, eleven were cohort studies and one was a retrospective report of a series of patients. The characteristics of the included studies, including descriptions of methods and results, are shown in Table 2, Table 3 and Table 4 for RPR treatment, lipotransfer, and other treatments separately.

### 3.1. Studies Assessing PRP Treatment

Of the thirteen studies reviewed, seven involved the use of PRP in the treatment of VLS.

Goldstein et al. [30] evaluated the efficacy of PRP in the treatment of lichen sclerosus in a study involving 12 patients. Seven of them showed a reduction in inflammatory infiltration on histopathological examination, three showed no change and two showed a slight increase in inflammation. At the same time, the subjective symptoms reported by the patients decreased. No adverse effects were reported.

In 2019, Goldstein et al. [31] conducted another study—a randomized, double-blind, placebo-controlled trial. It involved 29 patients diagnosed with VLS. Nineteen women received PRP, while ten received the placebo. Among the patients treated with PRP, five showed improvements in inflammation on histopathological examination, ten showed no change, and four worsened. In the placebo group, an improvement was also observed in five patients, no change in four patients, and worsening in one patient. The only adverse effect associated with PRP therapy was the occurrence of bruising at the injection site.

Behnia-Willison et al. [28] applied three series of PRP treatments to patients with VLS. A reduction in skin lesions was observed in the majority of cases, while eight of twenty-eight patients achieved complete resolution of their lesions. More than half of the study participants reported the complete resolution of subjective symptoms such as soreness, dyspareunia, or discomfort. Importantly, 82.1% of the patients did not require further use of topical steroids after treatment.

Tedesco et al. [34] in 2022 conducted a study involving 51 women who were given three doses of PRP at 15-day intervals. Assessments were made before treatment and 6 months after treatment. A statistically significant reduction in subjective symptoms such as pain, burning, and itching was reported by patients. A moderate reduction in dyspareunia was also observed.

In the study by Tedesco et al. conducted in 2021 [33] involving six patients, three PRP series were administered at 15-day intervals. Patients underwent a clinical assessment using video thermography before treatment, seven days after the last injection, and 30 days after the end of therapy. The control group consisted of six healthy female volunteers. Before treatment, at least one hypothermic area was detected in each patient with VLS. After the end of therapy, 10 of the 12 previously identified foci showed significant reductions.

Medina Garrido et al. [32] studied 23 women with an unsatisfactory response to previous treatment with topical steroids. Patients underwent three sessions of PRP treatment and were assessed one month after each injection and then six and twelve months after the end of therapy. Significant reductions in subjective symptoms such as itching, burning, pain, and feelings of skin tightness were noted at all time points.

In 2024, Boero et al. [29] conducted a study using PRP derived from umbilical cord blood. This type of PRP, compared to autologous PRP, contains higher concentrations of anti-inflammatory molecules. Statistically significant improvements in both subjective symptoms and the clinical picture of the vulva were observed in all nine participants in the study.

### 3.2. Studies Assessing Lipotransfer Treatment

Of the thirteen studies reviewed, three involved the use of lipotransfer in the treatment of VLS.

Almadori et al. [35] conducted a study involving 33 women, 16 of whom were using concomitant topical steroid preparations and 17 of whom were not receiving additional treatment. All participants were postmenopausal. After adipose tissue transplantation containing ADSCs and progenitor cells, improvements were observed in all clinical parameters assessed. There was a significant reduction in fibrosis and an improvement in the architecture of the lesions. Importantly, both the use of topical treatment and the hormonal status of the patients did not affect the efficacy of the therapy. The authors confirm the high efficacy and safety of lipotransfer in the treatment of VLS.

Boero et al. [36] used autologous adipose tissue transplantation obtained from the abdominal or thigh area. In 94% of the patients, improvements in skin and mucosal atrophy were observed in the macroscopic evaluation. Microscopic examination showed reductions in hyperkeratosis, fibrosis, and chronic inflammation in the majority of participants. Quality of life indicators assessed by the Dermatology Life Quality Index (DLQI) and Female Sexual Function Index (FSFI) improved significantly after treatment.

Monreal [37] conducted a retrospective analysis of 39 patients who underwent autologous adipose tissue transplantation enriched with a stromal vascular fraction (SVF) containing stem cells. A clinically significant improvement in subjective symptoms was observed in 94.87% of participants. Only two patients felt the need to reapply clobetasol ointment at a later follow-up.

### 3.3. Studies Assessing Combined Treatment: PRP and Lipotransfer Treatments

Of the thirteen studies reviewed, one involved the use of combination therapy in the treatment of VLS.

Casabona et al. [38] conducted a retrospective analysis of 72 patients who had previously failed to achieve a satisfactory response to topical corticosteroid treatment. Each woman underwent a regenerative procedure consisting of the simultaneous administration of autologous PRP and fat grafting. The number of treatments performed depended on the individual clinical response and averaged four (median). After treatment, a significant reduction in subjective symptoms such as burning and itching was observed. Analysis of questionnaires assessing quality of life showed a statistically significant improvement after treatment. Upon physical examination, improvements in elasticity, thickening of vulvar tissues, and increased vascularization were noted, indicating a positive regenerative effect of the combination therapy.

### 3.4. Studies Assessing Other Substances Delivered by Mesotherapy

Of the thirteen studies analyzed, two involved combination therapy of VLS using other active substances administered by the mesotherapy technique.

In a study by Gkouvi and Gregoriou [39], three patients were involved who had previously been treated with topical clobetasol propionate for six weeks without improvement. They then underwent three mesotherapy treatments with 100 mg of micronized sterile collagen type I of heterologous origin dissolved in 4.5 mL of 0.9% NaCl solution with 0.5 mL of lidocaine. As early as after the second treatment session, subjective symptoms disappeared, and after the third treatment session, complete resolution of vulvar skin lesions was observed. No treatment-related adverse effects were reported.

Tedesco et al. [40] conducted a study in which hyaluronic acid was injected directly into vulvar skin lesions in 19 female patients. After treatment, a statistically significant improvement in the participants’ quality of life was observed. In three women, the skin lesions disappeared completely, which was confirmed by video thermography. The remaining patients, who also reported an improvement in subjective symptoms, showed a significant reduction in hypothermic areas on imaging.

## 4. Discussion

Our review identified 13 studies that used mesotherapy with various substances, namely, PRP, lipotransfer, collagen, and hyaluronic acid, to alleviate the symptoms of VLS in women. Treatments were applied using differentiated schedules with one to several sessions at different time intervals. To assess the effectiveness of treatments, clinical symptoms were rated combined with scales dedicated to evaluating VLS, quality of life, and sexual functioning. Histopathology examinations and thermography were also used. The evaluated studies showed the promising efficacy of PRP in the treatment of VLS, with a considerable reduction in subjective symptoms such as dyspareunia, pruritus, burning, or pain [29,30,32,34,38,40]. At the same time, improvements were observed in skin lesions assessed by physical examination, histopathology, and video thermography, including a partial or complete regression of lesions [31,33,40]. However, there was noticeable heterogeneity in the patient population and treatment schedules among the analyzed studies.

**Figure 3:**
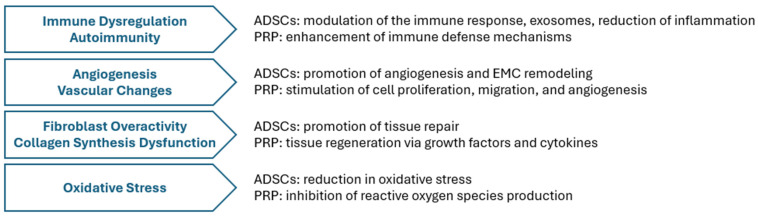
Juxtaposition of ADSCs’ and PRP’s mechanisms targeting VLS pathogenesis.

The mechanism of action of PRP is related to the presence of growth factors and cytokines that stimulate tissue regeneration and repair [41,42,43]. An important advantage of autologous PRP is its safety—the use of the patient’s own blood components minimizes the risk of side effects and immunological reactions. A certain alternative is homologous PRP [38,44]. Boero et al. [29] presented the results of a study using PRP derived from umbilical cord blood. It was shown to contain higher concentrations of anti-inflammatory factors compared to autologous PRP, potentially resulting in a more favorable clinical response. However, the use of donor-derived PRP carries a higher risk of complications and requires further studies [15]. Adipose tissue-derived stem cell therapies have also shown beneficial effects on the course of the disease, especially in terms of reducing fibrosis and inflammation within the vulvar tissues [45]. Improvements in subjective symptoms were also observed. These cells, classified as mesenchymal stem cells (MSCs), are characterized by their plasticity, differentiation capacity, and regenerative properties. They have a direct effect on immune cells and regulate cytokine secretion, which may explain their beneficial effect on the course of vulvar lichen sclerosus [15,46]. These new therapies, thanks to their regenerative, anti-inflammatory, and immune response-modulating properties, can contribute to significant improvements in both subjective symptoms (pruritus, burning, and dyspareunia) and vulvar tissue regeneration, as reflected in clinical and histopathological studies. PRP contains numerous growth factors and cytokines that stimulate repair processes, while ADSCs influence the inflammatory microenvironment by regulating immune cell activity. Although the extraction of biological material, either blood or adipose tissue, involves invasive procedures, both methods have a good safety profile, especially when autologous material is used. Homologous PRP, despite its potentially stronger anti-inflammatory effect, is associated with a higher risk of immune reactions and requires further clinical evaluation. Regenerative therapies are complemented by active substances applied by mesotherapy techniques, such as type I collagen and hyaluronic acid. Preliminary clinical reports suggest that these preparations can effectively support the reconstruction of the structures of the dermis and vulvar mucosa, leading to a reduction in lesions and an improvement in patients’ comfort. Collagen, a structural protein, plays a key role in maintaining tissue integrity, while hyaluronic acid promotes skin hydration and elasticity, with additional anti-inflammatory effects [47,48]. The modes of action of ADSCs and PRP, which target the pathophysiological mechanisms of VLS, are illustrated side by side in Figure 3.

VLS manifests itself by the presence of white lesions usually located on the labia minora, the clitoral frenulum, and the vaginal vestibule. The most common symptoms reported by patients include itching, dyspareunia, and a burning sensation. According to current recommendations, the first-line treatment remains high-potency topical corticosteroids, while calcineurin inhibitors may be used if there is no clinical response. In situations of resistance to local treatment, phototherapy or systemic immunosuppression, including cyclosporine or methotrexate, is considered [2]. However, a significant proportion of patients still experience a limited response to treatment and disease recurrence. Combined with the impacts of VLS on quality of life and sexual function, these make management particularly challenging. As a result, growing attention is being directed toward biological and regenerative therapies, such as PRP injections, ADSC grafting, and the use of active substances delivered through mesotherapy techniques. Our review reflects this emerging trend. In addition to studies focused exclusively on PRP or ADSCs, we identified research exploring other treatment protocols. One study evaluated the effectiveness of a combination therapy involving both PRP and stem cells, suggesting that their synergistic actions may offer enhanced clinical benefits, although current data remain limited [38]. Furthermore, two studies examined the use of alternative active substances delivered via mesotherapy for the treatment of lichen sclerosus. Gkouvi and Gregoriou [39] reported that type I collagen improved both subjective symptoms and visible skin lesions, while Tedesco et al. [40] found that hyaluronic acid administration led to a reduction in vulvar lesions.

Despite promising outcomes, the evidence presented in this review is limited by several methodological weaknesses. Most of the studies included were cohort designs, with only one RCT and one retrospective case series. The diversity of studied populations; variation in patient age; differences in treatment protocols, evaluation methods, and study endpoints; as well as short follow-up periods in most cases make it difficult to draw definitive conclusions on efficacy and tolerability. The absence of control groups in many studies further limits the strength of the evidence. To reliably determine the efficacy and safety of the regenerative therapies discussed, larger multicenter randomized studies with standardized protocols, consistent outcome measures, and extended follow-up periods are needed.

## 5. Conclusions

The use of PRP, ADSCs, and mesotherapy-delivered substances represents a promising adjunct treatment or alternative approach to standard treatments for VLS. Our review demonstrated their potential effectiveness in alleviating clinical symptoms, enhancing quality of life and sexual function, and improving tissue quality, as evidenced by thermographic and histopathological assessments. However, their clinical application may be limited by their invasive nature, the potential risk of immune reactions, and the absence of standardized treatment protocols. Current evidence is constrained by a small number of heterogeneous studies, often with limited sample sizes, a lack of control groups, and short follow-up durations. Future studies should prioritize large-scale, multicenter randomized controlled trials with extended follow-up, standardized intervention protocols, and objective outcome measures. Moreover, researchers should explore the molecular pathways targeted by these therapies, aiming to optimize their use and potentially combine them with conventional treatments to achieve durable remission and prevent disease progression.

## Figures and Tables

**Table 1:** Inclusion and exclusion criteria according to the PICOS methodology.

Parameter	Inclusion Criteria	Exclusion Criteria
Population	Women ≥ 18 years old with VLS	Women < 18 years old, men, other subjects, e.g., animals
Intervention/Exposure	Treatment with stem cells, PRP, or other mesotherapy products	Other types of treatment
Comparison	Not applicable	
Outcomes	Objective evaluation of treatment with stem cells, PRP, and other mesotherapy products	
Study design	RCT, non-RCTs, single arm, non-controlled	Not published in English Full version of the document not available Published before 2015 Literature reviews, book chapters, letters, editorials, notes, in vitro studies, case reports

PRP, platelet-rich plasma; RCT, randomized controlled trial; VLS, vulvar lichen sclerosus.

**Table 2:** Summary of studies evaluating PRP treatment.

Study	Patients *	PRP	Research Methods, Results, and Adverse Events
Behnia-Willison [28] 2016 Australian Cohort	N = 28 Age range: 22–28 years	PRP administered 2 times, 4–6 weeks apart, with an additional session at 12 months	Methods: colposcopy, verbal score for pain, Australian Pelvic Floor Questionnaire Results: After treatment, lesions were not seen in 8 (28.6%) patients, lesions became smaller in 17 (60.7%) patients, and lesions were the same in 3 (10.7%) patients (*p* < 0.001). In total, 8 (53.6%) patients had become asymptomatic, and 13 (46.4%) experienced intermittent symptoms (*p* < 0.001). After treatment (at 12 months or more), 23 (82.1%) of patients were no longer on steroids, while the remaining 5 (17.9%) used them sporadically (*p* < 0.001). Scores for pelvic floor disorders improved numerically, but the changes were not significant. Adverse events: Patients experienced minimal to moderate pain. On day 1, 26 patients (92.9%) reported pain scores between 2 and 3, while the remaining patients reported scores of 5 and 7. Adverse events, including infection, bleeding, and hematoma, were observed.
Boero [29] 2024 Italian Cohort	N = 9 Median age: 47 years Age range: 31–53 years	cord blood PRP; 1 unit of PRP per injection with >3 injections per patient per treatment; 3 treatments separated by 4–6 weeks	Methods: vulvoscopy; pruritus, burning, dyspareunia, and dysuria on NRS; FSFI; HADS; SF-12; Global Impression of Change questionnaire Results: All patients showed improved vulvar skin elasticity and nourishment, with no significant changes observed in the vulvar architecture. The reduction in the number of patients requiring maintenance TCSs was 55.5% (*p* = 0.014). Three months after treatment, five out of ten patients (55%, 95% CI: 24–76%) reported being either satisfied or very satisfied with the treatment, and the remaining four patients (44.4%) were uncertain. Seven of nine patients (78%) reported an improvement in their global impression of change. NRS scores were significantly decreased (*p* < 0.05). A significant improvement in sexual arousal and satisfaction (*p* < 0.05) was observed in all women who reported having a sexual partner both before and after treatment. The changes in the FSFI and HADS scores were not significant. Treatment tolerability: Moderate, with a mean NRS pain score of 4.4 (median 4; IQR 4–5) at needle insertion and 7.3 (median 7; IQR 6.75–8) for burning during PRP injection. Adverse events: No moderate/severe or unexpected adverse reactions.
Goldstein [30] 2017 USA Cohort	N = 12 Age not reported	PRP (5 mL) administered 2 times 6 weeks apart	Methods: biopsy, pruritus and vulvar burning on VAS’ change in IGA Results: A total of 7 (58%) patients had decreased inflammation on their post-treatment biopsies, 3 had no change, and 2 had a “minimal” increase in inflammation (*p* = 0.024). A significant change in IGA was observed (2.67 ± 0.49 vs. 1.83 ± 0.83; *p* = 0.0054). VAS scores for pruritus and burning did not change significantly. Adverse events: Transient discomfort and bruising at the biopsy and injection sites.
Goldstein [31] 2019 USA RCT	N = 29 19 on PRP, 10 on placebo Mean age 52.6 years	5 mL per treatment subdermally and intradermally; 2 PRP treatments 6 weeks apart	Methods: pretreatment and post-treatment biopsy, CSS Results: In the PRP group, histopathologic inflammation improved in 5 patients, did not change in 10 patients, and 4 worsened in 4 patients; in the placebo group, 5 patients had an improvement, 4 had no change, and 1 worsened (*p* = 0.542). The mean difference in the CSS patient domain between the initial and final visits was −7.74 for patients receiving PRP and −9.44 for patients receiving the placebo (*p* = 0.654). Adverse events: Bruising.
Medina Garrido [32] 2023 Cohort	N = 23 Mean age 66.6 years	PRP (4 mL) administered 3 times, 4 to 6 weeks apart	Methods: CSS Results: After the 3rd infiltration, a decrease in symptoms was observed: vulvar itching (6.8 vs. 3.9), burning (5.8 vs. 3.9), and soreness (4.1 vs. 2.5). Subjective improvement changed from 1.9 to 2.5. Adverse events: Mild to moderate pain after the procedure; no adverse outcomes (e.g., infection and bleeding) were reported.
Tedesco [33] 2021 Italian Cohort	N = 6 Age range: 46–58 years	PRP (4 mL) administered 3 times every 15 days	Methods: video thermography Results: At baseline, 12 hypothermic areas were identified. After 7 days post-treatment, 10 hypothermic areas were no longer recognizable, while 2 presented with increased temperature compared to baseline. After 30 days post-treatment, eight previously hypothermic areas remained undetectable, two became visible but showed an increased temperature, and the remaining two visible areas disappeared. Subjectively, all patients reported marked symptom improvement. Adverse events: Not reported.
Tedesco [34] 2022 Italian Cohort	N = 51 Age range: 22–84 years	PRP (4 mL) administered 3 times, every 15 days	Methods: physical assessment, interview, DLQI Results: In women, pain decreased from 33.3% to 7.8% (*p* < 0.0001), burning sensation decreased from 51% to 15.7% (*p* = 0.0001), itching decreased from 80.4% to 21.6% (*p* < 0.0001), and dyspareunia decreased from 37.3% to 31.4% (*p* < 0.25). The percentage of patients without symptoms increased from 14.9% at baseline to 52.1% after treatment. The median DLQI score decreased from 6 to 4. Adverse events: No adverse outcomes were reported.

* The number of patients who completed the study. Only female participants were included. DLQI, Dermatology Life Quality Index; FSFI, Female Sexual Function Index; HADS, Hospital Anxiety and Depression Scale; IGA, Investigator’s Global Assessment; NRS, Numerical Rating Scale; PRP, platelet-rich plasma; RCT, randomized controlled trial; SF-12, Short Form-12 item questionnaire; TCSs, topical corticosteroids; VAS, visual analog scale.

**Table 3:** Summary of studies evaluating lipotransfer treatment.

Study	Patients *	Lipotransfer	Research Methods, Results, and Adverse Events
Almadori [35] 2020 UK Cohort	N = 33 Mean age 50.5 ± 12.5 years	Autologous lipotransfer; centrifuged adipose tissue (1 mL) rich in ASCs and progenitor cells was administered as a single treatment	Methods: FSFI; FSDS; itching, burning, and soreness on VAS; PASS-20; HADS; RAS; WMQ-R; VASS Results: The mean (SD) follow-up was 12.9 (3.5) months. Sexual function improved after treatment (*p* < 0.001), as well as the distress associated with sexuality (*p* < 0.0001). Patients reported improvements in itching (*p* < 0.001), burning (*p* < 0.05), soreness (*p* < 0.001), and pain (*p* < 0.0001), as well as in romantic relationships (*p* < 0.05), anxiety (*p* < 0.0001), and depression (*p* < 0.0001). There was no significant improvement in self-care, as measured by the self-disgust assessment (*p* = 0.42). The treated patients presented overall improvements in dermal fibrosis and overall architecture. Adverse events: Not reported.
Boero [36] 2015 Italian Cohort	N = 36 Mean age: 54 years Age range: 25–80 years	Fat grafting: one to three procedures, depending on disease severity, symptom resolution, and patient satisfaction	Methods: clinical assessment, vulvoscopy, punch biopsy, DLQI, FSFI Results: A total of 34 (94%) patients reported better vulvar trophism of the skin and mucosae; 27 (75%) had improvements in the caliber and elasticity of the vaginal introitus; 18 (50%) reported a reduction in the clitoris burying degree; 30 (83%) had an increase in the volume of the labia majora and minora and experienced complete resolution of scratching-related lesions, and 28 (78%) exhibited an improvement leading to the disappearance of white lesions. Thirty-four patients (95%) discontinued the routine use of TCSs. The improvement in life quality was significant for both DLQI (*p* < 0.001) and FSFI (*p* < 0.001). Adverse events: No intra- and post-operative adverse events.
Monreal [37] 2020 Spanish Cohort	N = 39 Mean age: 45.79 ± 13.64 years	Autologous lipotransfer; ~15–20 mL of adipose tissue for fat grafting was mixed with 3.3–4.3 mL of an SVF cell suspension for subdermal and subcutaneous infiltration, and 1.5 mL of the SVF suspension was used for intradermal injection; single session	Methods: physical examination, photographs of the external genital area, VVSQ Results: A total of 37 (94.87%) patients improved at 6 and 24 months, and 2 (5.12%) patients worsened. The test results showed a global score at 24 months that was significantly lower than before treatment (43.59 ± 28.23 vs. 93.56 ± 33.97; *p* < 0.05). In the categories of symptoms, signs, social functioning, and sexual functioning, scores at 24 months (8.46 ± 7.28, 7.51 ± 5.96, 19.18 ± 18.36 and 8.44 ± 7.95) were significantly lower than those obtained before treatment (21.18 ± 9.51, 16.90 ± 8.71, 39.85 ± 20.09 and 15.64 ± 10.58; *p* < 0.05). Adverse events: All patients had mild genital discomfort and swelling resolving within six days, while donor site inflammation lasted up to six weeks. No complications or side effects occurred during the study.

* The number of patients who completed the study. Only female participants were included. DLQI, Dermatology Life Quality Index; FSDS, Female Sexual Distress Scale; FSFI, Female Sexual Function Index; HADS, Hospital Anxiety and Depression Scale; PASS-20, Pain Anxiety Symptoms Scale 20; RAS, Relationship Assessment Scale; SVF, stromal vascular fraction; TCSs, topical corticosteroids; VAS, visual analog scale; VASS, Vulvar Architecture Severity Scale; VVSQ, Vulvovaginal Symptoms Questionnaire; WMQ-R, Revised Wound Management Questionnaire.

**Table 4:** Summary of studies evaluating other treatments.

Study	Patients *	Treatment	Research Methods, Results, and Adverse Events
Casabona [38] 2023 Italy Cohort	N = 72 Age range: 26–75 years	Autologous PRP and fat grafting; repeated treatments were conducted as deemed necessary, adjusted to the clinical response	Methods: DLQI, Skindex-29, FSFI, CSS Results: Follow-up after the last surgery lasted for three months. After treatment, all scores improved: Skindex-29 (−31.8 [IQR: 42.1, −21.8] points; *p* < 0.001), FSFI (7.6 [IQR: 2.7, 14.7)] points; *p* < 0.001), patient-administered CSS (−24 [IQR: −30, −15] points; *p* < 0.001), DLQI (−9 [IQR: −17, −7] points; *p* < 0.001), physician administered CSS (−5 [IQR: −7, −5] points; *p* < 0.001), and IGA (median ΔIGA: −4, IQR: −4, −3; *p* < 0.001). Post-operative symptoms improved; burning and pruritus were reported by a smaller number of patients, in 69.4% and 76.4% of cases. Pruritus was moderate/severe in 83.3% of cases preoperatively and in only 2.7% of cases postoperatively. Similarly, burning was moderate/severe. All patient-administered questionnaires showed significant improvements after surgery in comparison with the baseline. Adverse events: Not reported.
Gkouvi [39] 2020 Grecian Case series	N = 3 Age: 36, 47, and 61 years	Heterologous type I collagen intradermally or subdermal; 4 treatments every 2 weeks, followed by maintenance treatment once every 2 months.	Methods: clinical assessment of lesions on VAS Results: The lesion surface area decreased after the first treatment and resolved completely after the third. Pruritus, soreness, discomfort, and dyspareunia improved by 50–75% in two patients and resolved in the third within 10 days of the first session. All patients were symptom-free 10 days after the second session, with no relapses during 12 months of maintenance therapy. Adverse events: All patients reported minimal to moderate injection pain (VAS < 5). No adverse events were reported.
Tedesco [40] 2023 Italian Cohort	N = 19 Median age: 58 years Age range: 21–78 years	Hyaluronic acid (2 mL; 32 mg/mL, 1:1 mix of high- [1100–1400 kDa] and low-molecular-weight [80–100 kDa]) administered intradermally once a month for 3 sessions	Methods: video thermography; intensity of itching, burning, pain, and dyspareunia; DLQI; FSFI; overall satisfaction Results: Significantly fewer patients presented with itching (17 vs. 5; *p* ≤ 0.001), pain (7 vs. 1; *p* = 0.031), and burning sensation (12 vs. 3; *p* = 0.004) at 6 months, but not with dyspareunia (16 vs. 12; *p* = 0.219) At baseline, all patients had at least one symptom, while at 6 months, 5 out 19 (26.31%) patients were without symptoms. Patients with FSFI scores > 26 increased from 5.3% at baseline to 26.3% at 6 months (*p* = 0.084). The DLQI score changed from 5.89 ± 3.68 at baseline to 3.42 ± 2.36 at 6 months (*p* = 0.002). The increase in the temperature of the perineal area was correlated with better outcomes. Adverse events: No side effects during and after infiltrations.

* The number of patients who completed the study. Only female participants were included. DLQI, Dermatology Life Quality Index; IGA, Investigator’s Global Assessment; VAS, visual analog scale.

## Data Availability

Data supporting the findings of this study are available within the article.

## Abbreviations

The following abbreviations are used in this manuscript:

ADSC	Adipose-derived stem cells
CCS	Clinical Scoring System for Vulvar Lichen Sclerosus
DLQI	Dermatology Life Quality Index
FSDS	Female Sexual Distress Scale
FSFI	Female Sexual Function Index
HADS	Hospital Anxiety and Depression Scale
HCC	Hybrid cooperative complexes of hyaluronic acid
IGA	Investigator’s Global Assessment
NRS	Numerical Rating Scale
PASS-20	Pain Anxiety Symptoms Scale 20
PRP	Platelet-rich plasma
RAS	Relationship Assessment Scale
RCT	Randomized controlled trial
SF-12	Short Form-12 item questionnaire
SVF	Stromal vascular fraction
TCSs	Topical corticosteroids
VAS	Visual analog scale
VASS	Vulvar Architecture Severity Scale
VLS	Vulvar lichen sclerosus
VVSQ	Vulvovaginal Symptoms Questionnaire
WMQ-R	Revised Wound Management Questionnaire
